# The effect of parental ABO blood group on fetal surveillance

**Published:** 2013-10-22

**Authors:** E Abdollahi, F Tavasolian, N Ghasemi, M Vakili, A Amini

**Affiliations:** 1 Department of Immunology, Faculty of Medicine, Shahid Sadoughi University of Medical Sciences, Yazd, Iran.; 2 Medical Genetics, Research and clinical Centre for Infertility, Shahid Sadoughi University of Medical Sciences and Health Services, Yazd, Iran.; 3 Department of Community Medicine, Faculty of Medicine, Shahid Sadoughi University of Medical Sciences, Yazd, Iran.; 4 Research center of reproductive immunology, School of paramedical sciences, Shahid Sadoughi University of Medical Sciences, Yazd, Iran.

**Keywords:** ABO blood group system, fetal, surveillance

## Abstract

**Background:**

Several factors may cause infertility and fetal loss. Blood groups antigens seem to be implied in the mechanisms of infertility and fetal loss. Maternal natural antibody can react against father’s blood group antigens on spermatozoa. The effects of parental blood group system on infertility and fetal surveillance perceived by its manifestation in prezygotic (caused infertility) and postzygotic (caused fetal loss) stages. Objective of the present study is to determine the effect of parental ABO blood group on fetal surveillance and men infertility.

**Materials and Methods:**

This is a retrospective, cross sectional study. Our study was carried out in fertility and infertility center of Yazd city. Blood group of 118males (group1:100 males with infertility and group 2: 18 males with abortion history in female partners) that referred to this center was evaluated based on medical document’s patients.

Data were analyzed with SPSS 16 software using chi-square test. The results were considered significant when P-value was <0.05, CI: 0.95.

**Results:**

Results indicated that overall distribution of blood groups in group 1 was:50%, 25%, 16% and 9% and in group 2: 56%,27%,11%,6% for blood groups O, A, B and AB respectively. There is a significant relationship between male infertility and blood group O (P value = 0.01). There is also a significant relationship between parental blood group O and fetal loss in group 2 (P value =0.03)

**Conclusion:**

The present study revealed that there is a significant relationship between father’s blood group O and fetal loss, so that appropriate intervention strategies can be followed.

## Introduction

Karl Landsteiner was the first person to find the ABO blood group system in 1901. According to the international society of blood transfusion (ISBT), there are about 250 blood antigens which have been divided into 29 blood group systems(1,2).The ABO blood group antigens is considered as the clinically significant blood group system.After the discovery of an association between stomach cancer and blood type A in 1953, there have been several studies on possible relationship of blood types to certain diseases(1,2). In addition importance of ABO blood groups in blood transfusion, the ABO blood groups has been associated with several diseases (Table I). It is possible that there is a relationship between type of blood group and men infertility. Recent works demonstrate that spermatozoa have detectable blood group antigens. The presence of group-specific substances in the female’s cervical secretions may prevent fertilization (3). Meiotic drive, in which certain sperm from a heterozygous male are favored for fertilization because of their blood group, may also be significant(4). But once fertilization has occurred, the mother’s natural antibody reacts against father’s blood group antigens on spermatozoa. Theoretically, the fertilized zygote should have a definable blood group. If there is sufficient antigenic stimulus, the mother’s antibody titer will determine the degree and time of the reaction. A higher titer may prevent fertilization, a lower titer (but one that is still relatively high) may cause loss of the fetus at an early age(5). The aim of this study is to determine relationship between ABO blood group and male infertility.In addition to, we want to evaluate possible relationship between parentalblood groups and fetal losses in female partners.

## Materials and Methods

This is a retrospective, cross sectional survey. This study was carried out in fertility and infertility center of Yazd city. Data was collected by census method during 2012 to 2013. Patients consisted of 118men divided two groups: group 1, 100 infertile men withoutabortion history in female partnersandgroup 2, 18 infertile men with abortion history in femalepartners. Female partnerswere withoutanatomical, microbial, viral, genetical disease.Hormone profile tests and tests for ovulation and tubal patency of Female partners were normal. In other hand,according medical evidences, female partners werehealthy and fertile. Male partners(studied patients) with abnormal investigations were included in the study. The investigations includedsemen analysis. A detailed sexual, occupational, medical, surgical history and abortion history in female partners was recorded. Because the these patients were from all over the Iran ,so the distribution of ABO blood group in infertile males was compared with statistically information provided by Iran Blood Transfusion Organization. 

**Statistical Analysis**

Statistical analysis was done by SPSS16 software using chi-square test for comparison of pair variables. P values <0.05 were considered significant (CI: 0.95%).

## Results

The mean age of infertile men without abortion history in female partners (group 1) was 37.6 (24 to 49) and The mean age of men with abortion history in female partners(group 2)was 41 (19 to43). The difference was not significant (Pvalue=0.33). Overall distribution of ABO and Rh blood groups in group 1 and group 2 have been shown in TableII. According to Iranian Blood Transfusion Organization data(IBTO,Tehran), overall distribution of blood groups was 37.62%, 30.25%, 24.36%, 7.77% for blood groups O, A, B and AB respectively.Our results indicated that there is a significant relationship between male infertility and O blood group ( P value =0.01). Our results also indicated that there is a significant relationship between fetal losses and parental blood group O in group 2( P value =0.03). There is a significant relationship between negative Rh blood group and men infertility(P value=0.02).

**Table I:** ABO blood groups and diseases

**Disease associated**	**Type of associated risk**	**Blood group**
**Squamous cell carcinoma of skin** **(6)**	Low	O
**Pancreatic cancer** **(7,8)**	Low	O
**Ovarian cancer** **(9)**	High	B
**Gastric cancer** **(10)**	High Low	A O
**Breast cancer** **(11)**	High	O
**Cervix cancer** **(11)**	High	B &O
**Lung cancer** **(11)**	High	B
**Buccal cancer** **(11)**	High	B
**Cholera & GI infections by E.coli(12)**	High	O
**H.pylori infection &GI Ulceration** **(12)**	High	O
**Ischemic heart disease** **(13)**	High	AB
**Otitis media with effusion** **(14)**	Low	O

**Table II:** Percentage of blood groups

**Group**	**Type of blood group**	**Percentage of each blood group**	**Percentageof Rh blood group**	
			**positive**	**negative**
**Group 1**	A	25%	24%	1%
	B	16%	15%	1%
	AB	9%	9%	0%
	O	50%	44%	6%
	sum	100%	92%	8%
**Group 2**	A	27%	7%	6%
	B	11%	13%	0%
	AB	6%	7%	0%
	O	56%	60%	7%
	sum	100%	87%	13%
	P value	0.44	P value	0.04

**Figure 1:**
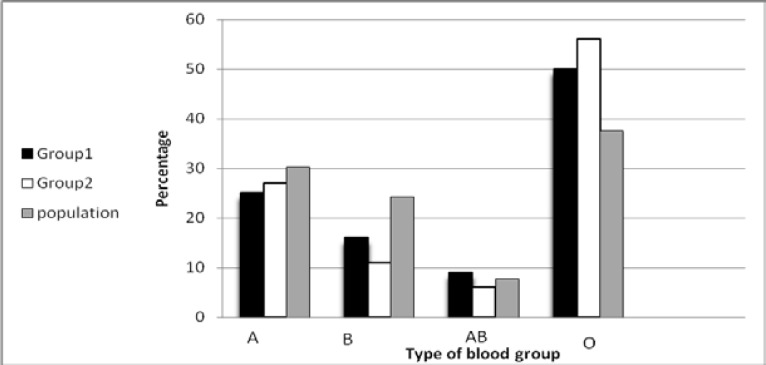
Distribution of blood groups and percentage

## Discussion

Many studies have reported the association of ABO blood groups with several diseases(6-14). This study was done to find any association of ABO blood groups with infertility.In the present study, there was a significant relationship betweenABO blood group andmen infertility. 0ur study showed,there is a strong relationship between paternal blood group O and fetal losses in female partners.Shoaib Khan et al found that blood group O is strongly related to male infertility(15). The basis for this finding may be the presence of seminal blood group antigens in secretors which could lead to antisperm antibodies and infertility.One research in America indicated that maternal blood group O was associated with fetal losses in 49.82%(16). In addition, another study in japan showed the frequency of deaths for fetuses bearing the A gene (genes) appeared lower than that for fetuses without it (17).

 Several studies have been carried out to evaluate the association of ABO incompatibility in couples with infertility. One survey showed 30-40% of infertile couples has ABO incompatibility and has concluded that ABO incompatibility between partners is a significant contributor to infertility (18).

Some studies indicated that there is no association between ABO blood groups with human fertility (19,20). 

Several studies conducted to find the association of ABO blood group antigens with anti sperm antibodies in infertile couples. These studies indicated that ABO blood group antigens do not significantly contribute to cervical antisperm antibody formation or infertility (21,22,23,24).

Another study has suggested that theremight be some measure of low zone tolerance to ABO antigens on spermatozoa and therefore ABO incompatibility might not significantly contribute to infertility (22).

Association of parental ABO blood group incompatibility with recurrent abortions is also controversial. Some studies found that blood group incompatibility is not an important etiology in the causation of spontaneous abortions(18,25). On the contrary, another research found that couples with recurrent spontaneous abortion had significantly higher incidence of parental ABO incompatibility than fertile couples (26).

Our study suggests that blood group O shows significant relationship with men infertility. Blood group O also shows significant relationship with fetal losses. These findings are in line with previous researches on subject of our study and confirm them. But generally, as a mentioned,there are some contrary results about relationship between ABO blood groups and fertility or infertility. It is possible; these differences caused by genetic diversity in different populations.Future studies can focus on other blood groups and infertility or fertility in men and women.

## Conclusion

The prevalence of male infertility in blood group O was invariably higher than in all other. There was the strong relationship between father’s blood group O and fetal loss in females’ partners. 
